# PRESIDENTIAL SYMPOSIUM: HIGHLIGHTING EMERGING PROFESSIONALS WHO BUILD BRIDGES, CATALYZE RESEARCH, AND EMPOWER ALL AGES

**DOI:** 10.1093/geroni/igad104.1071

**Published:** 2023-12-21

**Authors:** Brianna Morgan, Rita Hu, Jacqui Smith

## Abstract

There are many ways to carve a path forward in aging research, policy, and practice. Sometimes the career possibilities are overwhelming, leaving us unsure of how best to proceed. As aging professionals, we know that there is much to learn from those who have gone before us. Therefore, in the ESPO Presidential Symposium we highlight four emerging professionals who are trailblazing diverse and fulfilling career paths in aging by building bridges, catalyzing research, and empowering all ages. Speakers include Drs. Cal Halvorsen, Lindsay Kobayashi, Jamie Justice, and Glenna Brewster Glasgow. Dr. Jacqui Smith will serve as the discussant, synthesizing main findings, emphasizing positive outcomes, and exploring challenges. Starting with identifying the “So What?” of his career, Dr. Cal Halvorsen will explore how his practice roots in policy and advocacy led to his current approach of merging scholarly pursuits and public communication to make an impact beyond the walls of academia. Next, moving to cross-national aging research, Dr. Lindsay Kobayashi will discuss the opportunities and challenges of combining diverse cross-national cohorts to identify policy-relevant and context-specific modifiers of health risks for older adults. Next, Dr. Jamie Justice will focus on her career in translational geroscience and share a translational framework for aging outcomes trials. Finally, from an interdisciplinary approach to conducting research with families living with cognitive impairment in the US and the Caribbean, Dr. Glenna Brewster Glasgow will reflect on practical strategies to develop and maintain collaborative relationships with researchers and stakeholders locally, nationally, and internationally.

